# Photocatalytic Removal of the Greenhouse Gas Nitrous Oxide by Liposomal Microreactors

**DOI:** 10.1002/ange.202210572

**Published:** 2022-09-05

**Authors:** Samuel E. H. Piper, Carla Casadevall, Erwin Reisner, Thomas A. Clarke, Lars J. C. Jeuken, Andrew J. Gates, Julea N. Butt

**Affiliations:** ^1^ School of Chemistry University of East Anglia Norwich Research Park Norwich NR4 7TJ UK; ^2^ Yusuf Hamied Department of Chemistry University of Cambridge Lensfield Road Cambridge CB2 1EW UK; ^3^ School of Biological Sciences University of East Anglia Norwich Research Park Norwich NR4 7TJ UK; ^4^ Leiden Institute of Chemistry Leiden University PO Box 9502 2300 RA Leiden The Netherlands

**Keywords:** Carbon Dot, Enzyme Catalysis, Liposomes, Nitrous Oxide, Photochemistry

## Abstract

Nitrous oxide (N_2_O) is a potent greenhouse and ozone‐reactive gas for which emissions are growing rapidly due to increasingly intensive agriculture. Synthetic catalysts for N_2_O decomposition typically contain precious metals and/or operate at elevated temperatures driving a desire for more sustainable alternatives. Here we demonstrate self‐assembly of liposomal microreactors enabling catalytic reduction of N_2_O to the climate neutral product N_2_. Photoexcitation of graphitic N‐doped carbon dots delivers electrons to encapsulated N_2_O Reductase enzymes via a lipid‐soluble biomolecular wire provided by the MtrCAB protein complex. Within the microreactor, electron transfer from MtrCAB to N_2_O Reductase is facilitated by the general redox mediator methyl viologen. The liposomal microreactors use only earth‐abundant elements to catalyze N_2_O removal in ambient, aqueous conditions.

## Graphical abstract

Self‐assembled liposome microreactors mimic the spatial separation of redox reactions seen in respiration and photosynthesis. Catalytic reduction of the climate heating gas N_2_O to benign N_2_ by encapsulated enzyme is driven by irradiation of external carbon dots through an electron transfer pathway defined by a membrane‐spanning cytochrome and redox‐cycling of internal methyl viologen.

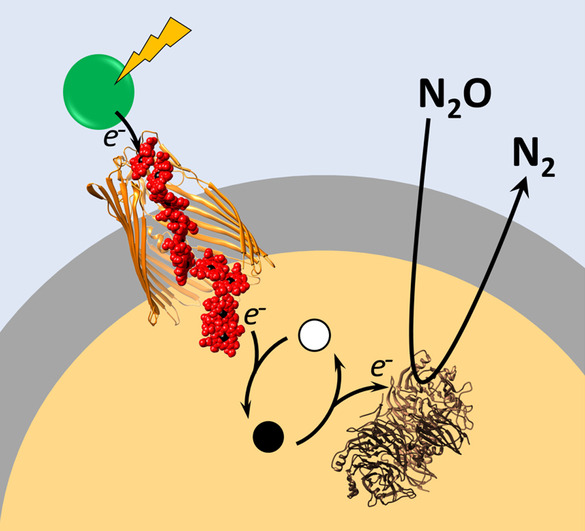

## Introduction

Artificial microreactors^[1]^ are attractive for potential applications across biotechnology and medicine, providing a route to greater understanding of biological compartmentalization, and supporting bottom‐up synthetic biology by providing a chassis for artificial cells. At their most basic, microreactors are composed of an interior compartment where reactions occur and a semi‐permeable shell through which reactants and products can pass. As a consequence much inspiration for the design of microreactors is provided by the cells and organelles of biology. The latter are defined by lipid bilayer membranes equipped with molecules to couple processes occurring in the aqueous solutions on opposite sides of those membranes. Prominent examples^[2]^ are found in photosynthesis and respiration where spatially separated redox reactions are coupled by trans‐membrane electron transfer to drive the endergonic cellular syntheses of ATP (adenosine triphosphate) and NADH (dihydro nicotinamide adenine dinucleotide).

Synthetic lipid bilayer membranes are attractive microreactor scaffolds that form spontaneously from amphipathic lipids by supramolecular self‐assembly in aqueous solution. The thickness of the hydrophobic core is approximately 35 Å, too wide for direct electron transfer at reasonable rates,^[3]^ and two approaches have been recognized to facilitate controlled electron transfer across such synthetic bilayers.^[4]^ In one approach freely diffusing redox‐active charge carriers such as methyl viologen (MV) permeate the bilayer. In its oxidized, doubly charged state (MV^2+^) this bipyridilium compound is colorless, highly water soluble and membrane impermeant.^[5]^ One‐electron reduction (*E*_m_ −440 mV, all potentials versus Standard Hydrogen Electrode) generates a strongly colored, stable, and singly charged radical cation (MV⋅^+^) with delocalized positive charge that is membrane permeable. To deliver trans‐membrane electron transfer, reduction of MV^2+^ in an aqueous phase produces MV⋅^+^ which can diffuse across lipid bilayers to be oxidized in a second aqueous compartment.

**Figure 1:**
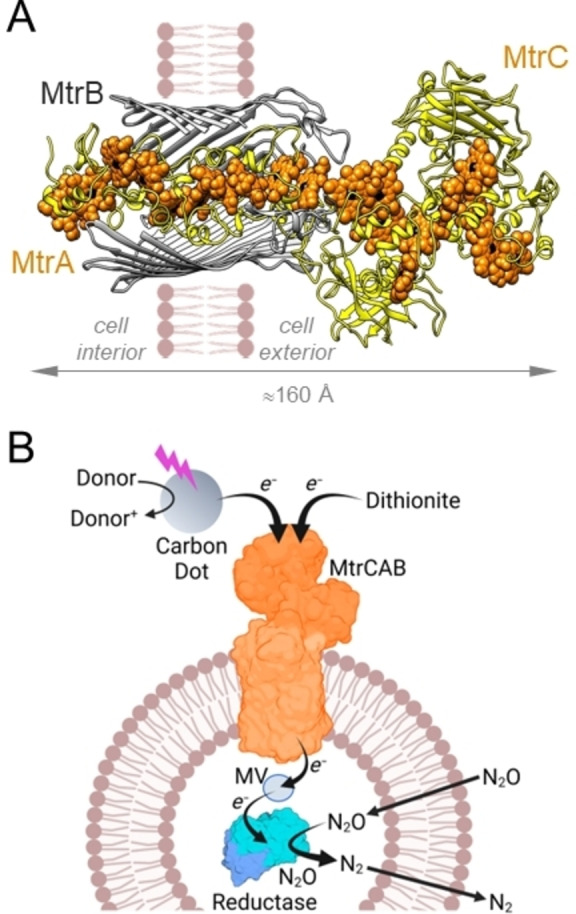
MtrCAB and its Role in Liposomal Microreactors for N_2_O Removal. A) Model^[15]^ for the MtrCAB complex from *S. oneidensis* based on the crystal structure^[7c]^ of *S. baltica* MtrCAB. Hemes (orange) with iron (black) are shown as spheres within the MtrA and MtrC proteins (yellow). The MtrA heme chain is insulated from the membrane by embedding within a beta‐barrel formed by MtrB (gray) for which the front surface is not shown. MtrA and MtrB are assembled as a naturally insulated biomolecular nanowire with both structural and functional attributes analogous to those of an electrical power cable. B) Schematic of a liposome microreactor with N_2_O Reductase encapsulated within a lipid bilayer membrane spanned by MtrCAB. Diagram not to scale and is purely to aid discussion, the orientation of MtrCAB is not experimentally defined. Panel B created with BioRender.com.

A second route to controlled electron transfer across lipid bilayers employs lipid‐soluble electron conduits, sometimes termed electron channels,^[4e]^ to span the bilayer. These systems typically position redox centers within the core of the hydrophobic membrane. This arrangement facilitates electron hopping between neighboring centers, and so across the bilayer, in several shorter and therefore faster steps. Such conduits may be synthetic single molecules or supramolecular assemblies.[^4c^, ^4d^, ^6^] Alternatively, electron transfer proteins can provide the conduit.[^4c^, ^4d^] An example is the MtrCAB complex^[7]^ of three proteins which performs bidirectional electron transfer across the outer membrane of *Shewanella* bacteria including *S. oneidensis* MR‐1 and *S. baltica* OS185. MtrCAB (Figure 1A) contains an electron transfer pathway of 185 Å defined by 20 close‐packed, redox‐active heme cofactors.^[7c]^

When purified and reconstituted into liposome bilayers,^[7a]^ MtrCAB performs fast transmembrane electron transfer in accord with the predicted electron transfer properties of this biomolecular wire.^[8]^ Building on those reports we describe here an artificial microreactor, illustrated schematically in Figure 1B, fitted with the MtrCAB nanowire to conduct electrons from external photoexcited carbon dots to an encapsulated redox enzyme. Our redox enzyme of choice was Nitrous Oxide (N_2_O) Reductase.^[9]^ This water‐soluble enzyme catalyzes the reductive decomposition of N_2_O: 

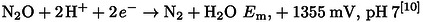




thereby converting the third most potent anthropogenic greenhouse gas^[11]^ and largest stratospheric ozone‐depleting substance to benign N_2_. Both gases can passively cross lipid bilayers thereby avoiding the need for dedicated transporters in our liposomal microreactors.

Increasingly intensive agriculture has underpinned a rise in global N_2_O emissions for each of the past four decades.[^11^, ^12^] While thermodynamically this molecule is a strong oxidant it is kinetically inert due to a large activation barrier to reaction.^[13]^ Indeed, N_2_O typically persists in the atmosphere for a century or more and this has significant consequences because N_2_O warms the atmosphere 300× more than the same mass of CO_2_ over such a period. Direct N_2_O decomposition is widely recognized as an attractive remediation technology^[12]^ but the most efficient synthetic catalysts include noble‐metals and/or operate best at elevated temperatures such that more sustainable alternatives are sought.[^12^, ^14^] The liposomal microreactors presented here enable photocatalytic reduction of N_2_O to the climate neutral product N_2_ under ambient conditions using only earth‐abundant elements.

## Results and Discussion

### Microreactor Assembly

Proteoliposomes with MtrCAB and encapsulated N_2_O Reductase were formed from a suspension of both proteins with the non‐ionic detergent octyl glucoside and *Escherichia coli* polar lipid extract (approximately 67 % phosphatidylethanolamine, 23 % phosphatidylglycerol, 10 % cardiolipin). Proteoliposome self‐assembly, as detailed in the Supporting Information, was driven by the addition of nonpolar, neutral macroporous polystyrene beads to adsorb detergent. The polystyrene beads were then allowed to settle, the solution recovered and proteoliposomes pelleted by ultracentrifugation. The supernatant was discarded and proteoliposomes resuspended in anaerobic 50 mM Tris:HCl, 10 mM KCl, pH 8.5. Further rounds of ultracentrifugation and resuspension were then performed until the supernatant was free of protein as confirmed by the Bradford assay. Dynamic light scattering (Figure S1) revealed monodisperse proteoliposomes of average hydrodynamic diameter 85±11 nm.

To assess how MtrCAB impacted on the properties of proteoliposomes prepared with N_2_O Reductase, equivalent samples were prepared by the protocol outlined above without the inclusion of MtrCAB. Monodisperse proteoliposomes were recovered with an average hydrodynamic diameter 146±25 nm (Figure S1). The impact of MtrCAB on liposome dimensions was most likely due to the influence of lipophilic MtrB (Figure 1A) on the packing of phospholipid headgroups and subsequent impact on bilayer curvature during liposome formation. Further investigation of this behavior was beyond the scope of this study. As described below, multiple lines of evidence confirmed that proteins included during proteoliposome formation were retained in the corresponding samples.

**Figure 2:**
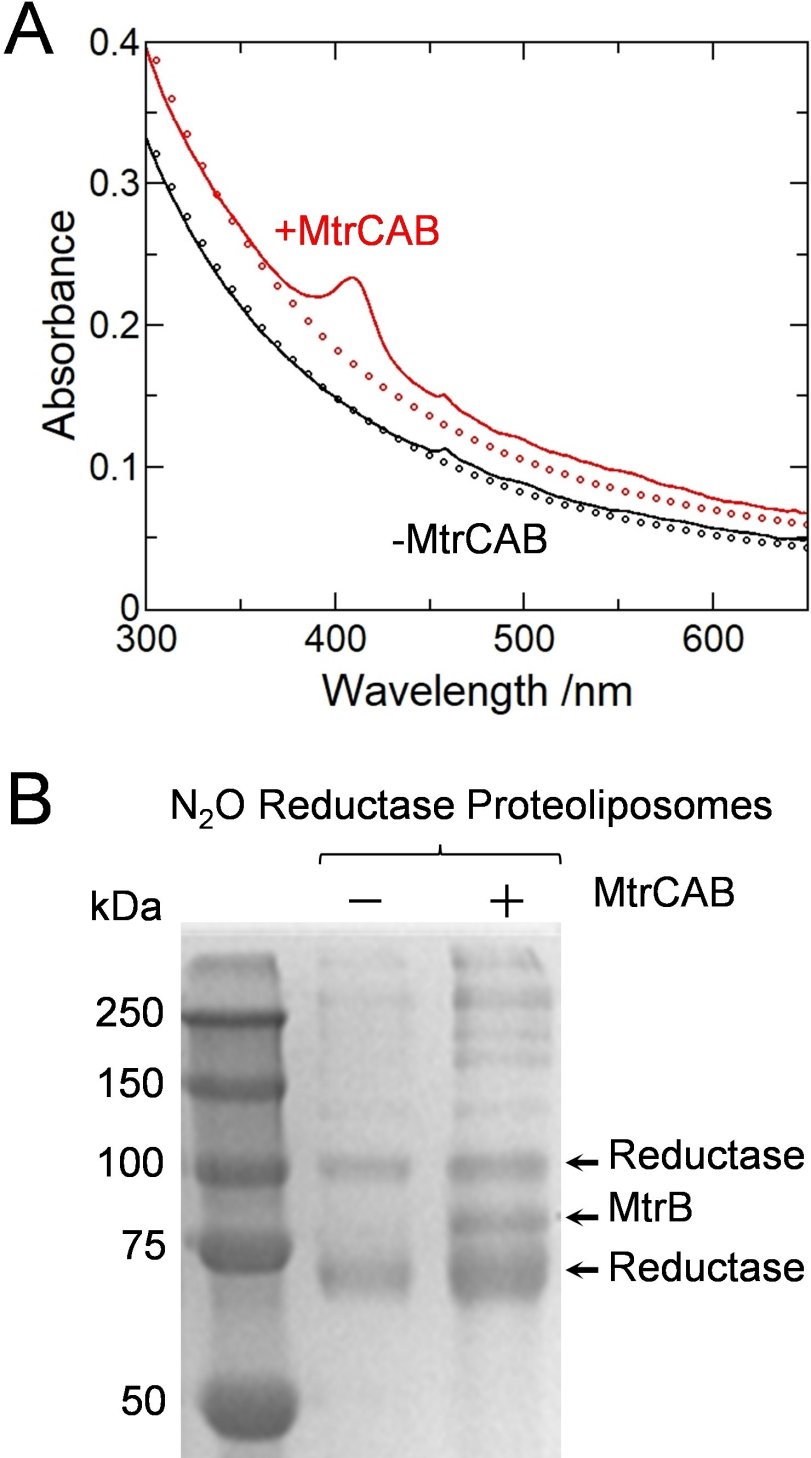
Characterization of Proteoliposomes. A) Electronic absorbance of N_2_O Reductase containing proteoliposomes with (red continuous line) and without (black continuous line)MtrCAB. Proteoliposomes (≈3 nM) in 50 mM Tris‐HCl, 10 mM KCl, pH 8.5. Circles show the estimated contribution to each spectrum from proteoliposome scattering, see Supporting Information for details. B) Coomassie stained SDS‐PAGE gel image for N_2_O Reductase containing proteoliposomes without (center lane) and with (right lane) MtrCAB. Molecular weight markers of the indicated mass (left lane).

The MtrCAB content was readily assessed by electronic absorbance spectroscopy. In the oxidized state MtrCAB contains Fe^III^ hemes that give a characteristic Soret band with maximum absorbance at 410 nm.[^7b^, ^15^] This feature was clearly present in spectra (Figure 2A red) of proteoliposomes prepared with MtrCAB and absent from spectra (Figure 2A black) of the corresponding control samples prepared without MtrCAB. After subtracting the spectral contribution from proteoliposome scattering, the concentration of MtrCAB was estimated by the Beer–Lambert law as 12 nM in a solution of approximately 3 nM proteoliposomes. From this we estimate approximately four MtrCAB complexes per liposome. This is consistent with zeta potential measurements for the proteoliposomes that gave values of −50±10 mV with no discernible dependence on the presence or absence of MtrCAB, which has a footprint of approximately 40 nm^2^ against an estimated liposome surface area of 23 000 nm^2^. Approximately 50 % of MtrCAB included in the protocol was incorporated into the proteoliposomes. This value is comparable to that achieved previously^[15]^ when proteoliposome formation was triggered by dilution to bring the octyl glucoside below its critical micelle concentration. The orientation of MtrCAB in the proteoliposome membranes is not known.

Denaturing polyacrylamide gel electrophoresis (SDS‐PAGE) confirmed that all desired proteins were present in the corresponding proteoliposomes. MtrB (75.5 kDa) was visualized by Coomassie stain as a band corresponding to protein of apparent mass ≈85 kDa (Figure 2B). The heme containing MtrA (38.6 kDa) and MtrC (75.0 kDa) proteins were more readily visualized by peroxidase‐linked heme stain as bands corresponding to proteins of apparent mass ≈33 and ≈75 kDa respectively (Figure S2). Also present in the Coomassie stained gel image (Figure 2B) are bands that reveal the presence of N_2_O Reductase (≈67 kDa). The bands corresponding to proteins of approximate mass 65 and 100 kDa can be assigned to monomer and dimer forms of this enzyme, respectively.

The catalytic activity of encapsulated N_2_O Reductase was confirmed after the proteoliposomes had been lysed to allow direct delivery of electrons to the enzyme from dithionite reduced MV^2+^ (Figure S3A). Lysis was triggered by the presence of 0.5 % (v/v) of the non‐ionic surfactant Triton X‐100. MtrCAB containing samples performed catalysis at a rate of 2.2±0.02 nmol N_2_O reduced min^−1^ (μL proteoliposome solution)^−1^. For samples without MtrCAB, the rate was lower 0.7±0.01 nmol N_2_O reduced min^−1^ (μL proteoliposome solution)^−1^. This behavior may indicate less enzyme encapsulated in the corresponding liposomes in line with the lower protein content detected by SDS‐PAGE (Figure 2B). Nevertheless, these assays clearly demonstrate that N_2_O Reductase had retained its activity through the process of encapsulation in proteoliposomes prepared with and without MtrCAB. In the absence of N_2_O Reductase the assay provides no evidence for MV⋅^+^ oxidation (Figure S3A) in accord with the stability and chemical inertness of N_2_O.

### Microreactor Catalysis

Having assembled the desired proteoliposome microcompartments we established conditions for N_2_O removal driven by photoexcitation of external carbon dots (Figure 1B). As described below, direct insight into reaction rate and mechanism came from electronic absorbance spectroscopy of samples containing sodium dithionite as external chemical reductant. N_2_O removal driven by irradiation of graphitic N‐doped carbon dots was then characterized by gas chromatography.

Sodium dithionite is a reductant previously shown to reduce the hemes of MtrCAB.[^7a^, ^7b^, ^16^] Electronic absorbance spectra (Figure S4A) revealed an immediate red‐shift of the heme Soret band maximum from 410 to 420 nm indicative of heme reduction from the Fe^III^ to the Fe^II^ state[^7b^, ^15^] following addition of sodium dithionite (100 μM) to anaerobic sealed cuvettes containing 750 μM N_2_O and 3 nM proteoliposomes. The spectra also revealed a strong absorbance band centered at 315 nm arising from sodium dithionite.^[17]^ That feature remained essentially unchanged over 20 min (Figure S4A). Thus, dithionite was not oxidized to sulfite (S_2_O_4_^2−^+2 H_2_O→2 HSO_3_^−^+2 *e* ^−^+2 H^+^; *E*_m_≈−500 mV, pH 7^[18]^) by coupled N_2_O reduction and our interpretation was that little or no electron transfer had occurred from chemically reduced MtrCAB to N_2_O Reductase.

**Figure 3:**
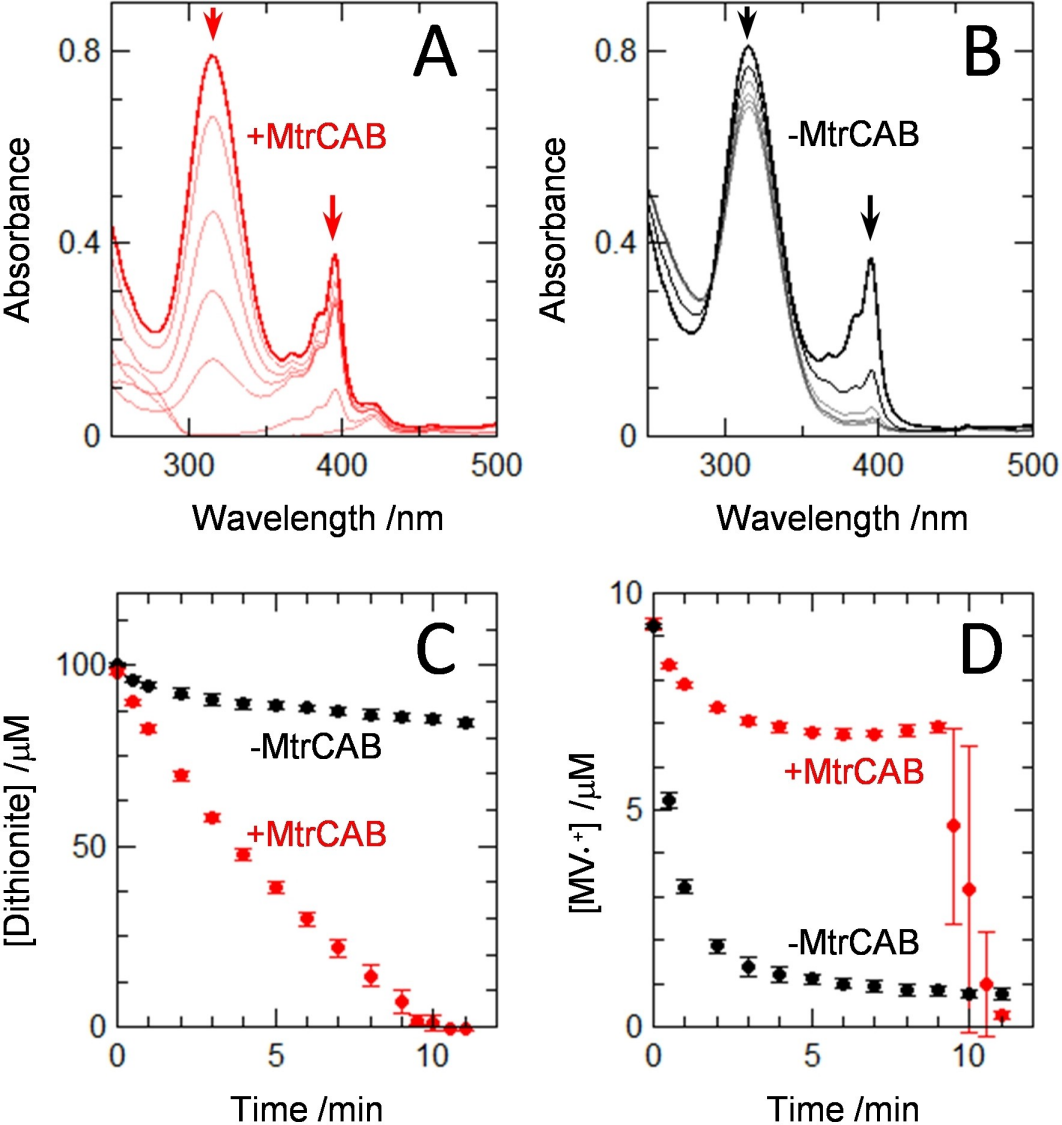
Dithionite‐Driven Proteoliposome N_2_O Reductase Activity. Electronic absorbance spectra for suspensions of N_2_O Reductase containing proteoliposomes with (A) and without (B) MtrCAB measured for 12 min after addition of 750 μM N_2_O at *t*=0 min. Arrows indicate the direction of spectral change for the features corresponding to sodium dithionite (315 nm) and MV⋅^+^ (395 nm). For (A) spectra at t = 0 (thick line), 1, 3, 5, 7, 10 and 11 (thin lines) min. For (B) spectra at t = 0 (thick line), 1, 3, 7, 8, and 11 (thin lines) min. Proteoliposomes (≈3 nM) in anaerobic 100 μM dithionite, 10 μM MV, 50 mM Tris‐HCl, 10 mM KCl, pH 8.5. Spectra are presented after subtraction of scattering due to proteoliposomes; see Supporting Information for details. Time course for oxidation of dithionite (C) and MV⋅^+^ (D) by N_2_O Reductase containing proteoliposomes with (red) and without (black) MtrCAB. In the presence of MtrCAB, after ≈9 min the dithionite is depleted which results in rapid oxidation of MV⋅^+^. Data are the average of *n*=3 datasets with error bars as standard deviation.

We reasoned that electron transfer from MtrCAB to encapsulated N_2_O Reductase should be enhanced if MV was present in the liposome interiors as a trace mediator of electron transfer. MV⋅^+^ is well described as an effective electron donor to N_2_O Reductase, see above and e.g., ref. [19]. The hemes of MtrCAB are redox active between 0 and −400 mV^[7b]^ and have been previously shown[^7a^, ^16^] to catalyse reduction of liposome entrapped MV^2+^ to MV⋅^+^. Thus, a small amount of MV^2+^ (10 μM) was added to previously prepared proteoliposomes with the expectation that the MV⋅^+^ formed on dithionite addition would rapidly cross the lipid bilayers, enter the liposomes and mediate electron transfer from MtrCAB to encapsulated N_2_O Reductase. Indeed, rapid bleaching of the dithionite absorbance at 315 nm (Figure 3A,C) was observed for proteoliposomes hosting MtrCAB and N_2_O Reductase when both MV and N_2_O were present. By contrast, parallel experiments for suspensions of proteoliposomes without MtrCAB showed almost no dithionite oxidation (Figure 3B,C).

Rates of dithionite‐dependent microreactor driven N_2_O reduction in the presence and absence of MtrCAB were 1.00 and 0.08±0.04 nmol N_2_O reduced min^−1^ (μL liposome stock)^−1^, respectively. The ten‐fold higher rate in the presence of MtrCAB is far more than can be explained by the slightly higher N_2_O Reductase activity of the MtrCAB containing liposomes (see above). Thus, we concluded that the MtrCAB protein complex provides the primary route for electron transfer across the bilayer to access the interior of the proteoliposome.

**Figure 4:**
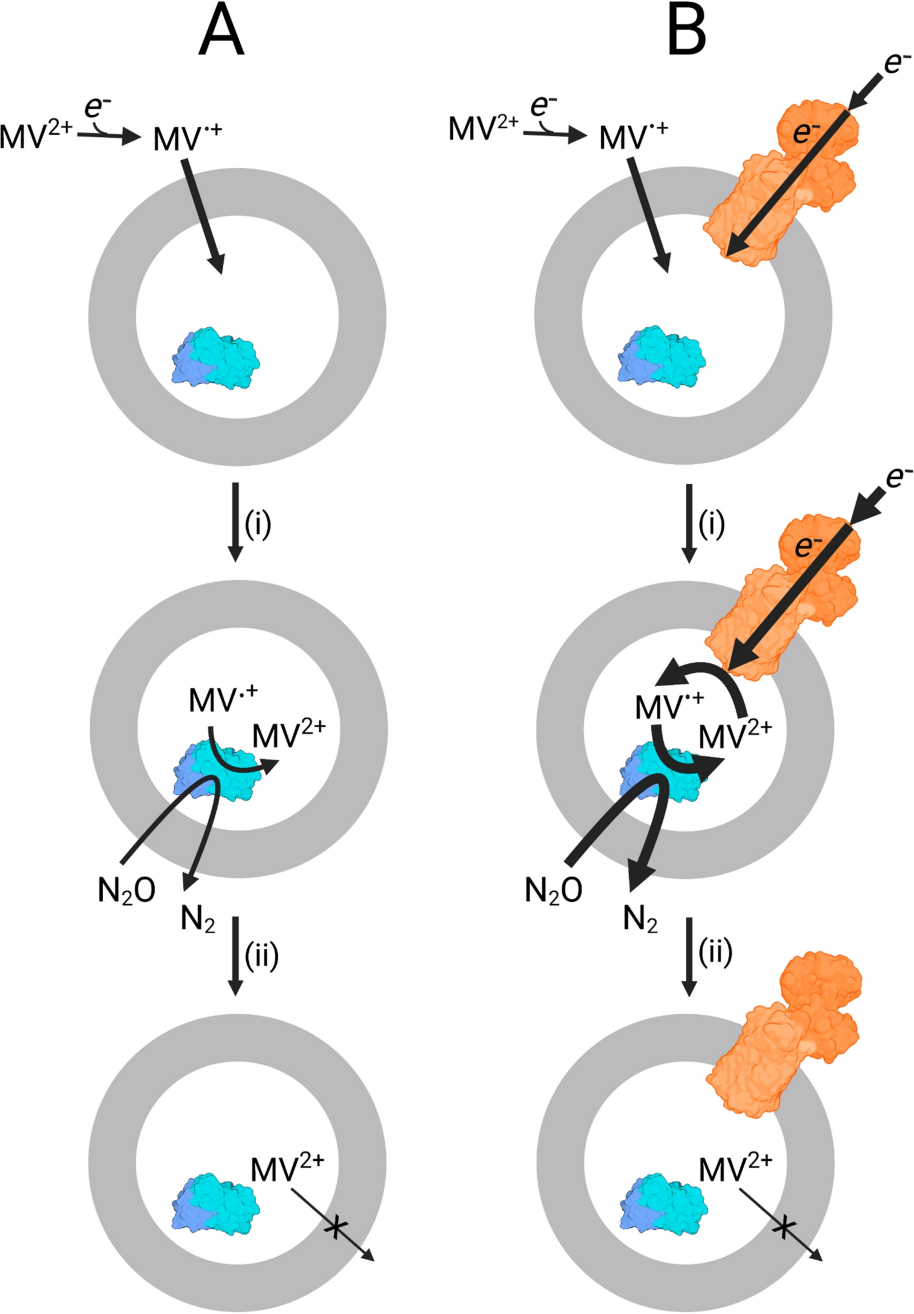
The Role of Methyl Viologen (MV) in MtrCAB Supported Proteoliposome N_2_O Reduction. Dithionite or irradiated carbon dots reduce MV^2+^ to bilayer permeable MV⋅^+^. Inside the proteoliposome MV⋅^+^ driven N_2_O reduction is catalyzed by N_2_O Reductase (blue) regenerating MV^2+^. A) In the absence of MtrCAB the MV^2+^ is trapped inside the liposome. B) In the presence of MtrCAB (orange) electrons from external (photo)reductants enter the liposome via the protein biowire and re‐reduce encapsulated MV^2+^. This process drives further N_2_O reduction. With N_2_O in excess of dithionite, when the latter becomes fully oxidized the MV is converted to MV^2+^ trapped inside the liposomes. Diagram not to scale and is purely to aid discussion, the orientation of MtrCAB is not experimentally defined. Created with BioRender.com.

Our proposed pathway for electron transfer in the liposomal microreactors is also supported by the behavior of the spectral feature centered on 395 nm (Figure 3A,B) that arises from MV⋅^+^.^[5b]^ With MV^2+^ introduced to the outside of the proteoliposomes the spectral data reveal rapid conversion to MV⋅^+^ due to excess sodium dithionite (Figure 3D). MV⋅^+^ can enter the proteoliposomes with relative ease^[5]^ and donate electrons to the encapsulated N_2_O reductase as illustrated schematically in Figure 4. The reoxidation product, MV^2+^, is then trapped inside the liposomes due to its higher charge. In the absence of MtrCAB the MV⋅^+^ concentration falls rapidly to a negligible level (Figure 3D black). This is because the internal MV^2+^ and external dithionite pools are insulated from one another on the timescale of these experiments (Figure 4A). In the presence of MtrCAB, the internal MV^2+^ is re‐reduced by electrons supplied from external dithionite via the MtrCAB electron conduit (Figure 4B). This process maintains MV⋅^+^ in steady state (Figure 3D red) at a concentration indicative of electron transfer from MtrCAB to MV^2+^ being faster than that from MV⋅^+^ to N_2_O Reductase. When dithionite has been depleted, after approximately 9 min, MV⋅^+^ becomes rapidly oxidized through electron transfer to the excess of N_2_O catalyzed by N_2_O Reductase (Figure 4B). The turnover number (TON) for MV is 20 and limited by the complete consumption of dithionite in these experiments.

To demonstrate photocatalytic removal of N_2_O by the liposomal microreactors we replaced sodium dithionite with irradiated graphitic N‐doped carbon dots as the external source of electrons. These photosensitizer nanoparticles^[20]^ have a negative surface charge and a diameter of 3.1±1.1 nm (Figure S5). They readily photoreduce MV^2+^ when irradiated with white light in the presence of EDTA (ethylenediaminetetraacetic acid) as sacrificial electron donor.^[20]^ The nanoparticles also catalyze light‐driven transmembrane electron transfer through MtrCAB.[^15^, ^21^]

**Figure 5:**
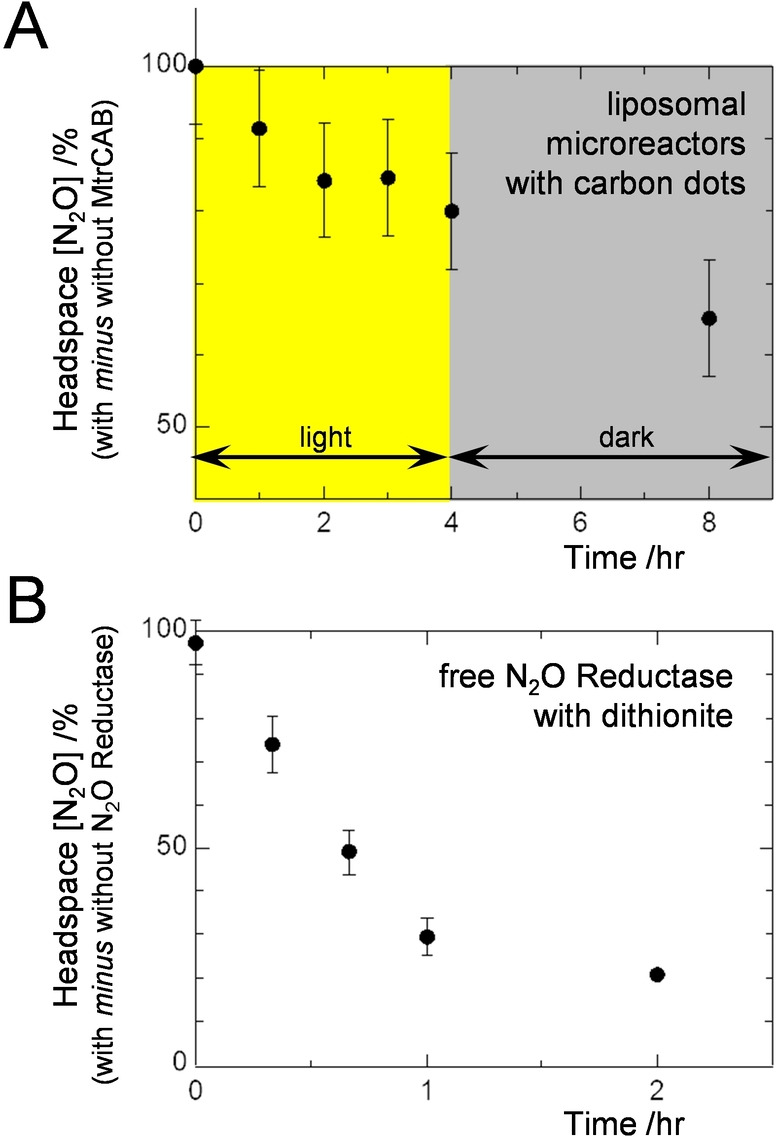
Gas Chromatographic Analysis of N_2_O Reduction. The difference in headspace N_2_O concentration is presented for A) suspensions of N_2_O Reductase containing proteoliposomes with and without MtrCAB, and B) solutions with and without free N_2_O Reductase. A) Anaerobic vials with N_2_O (1.5 μmol total) in 1 mL headspace and 2 mL of 100 μg mL^−1^ graphitic N‐doped carbon dots, 10 μM MV, 3 nM proteoliposomes, 25 mM EDTA, 50 mM Tris‐HCl, 10 mM KCl, pH 8.5. Proteoliposomes (8 nM) were introduced at *t*=0 hr. Irradiation with visible light (2.5 kW m^−2^) for 4 hr was followed by 4 hr in dark. Circles show the average of *n*=3 datasets with error bars as standard deviation. B) Anaerobic vials with N_2_O (1.5 μmol total) in 1 mL headspace and 2 mL of 1600 μM MV, 800 μM dithionite, 50 mM Tris:HCl, 10 mM KCl, pH 8.5. N_2_O Reductase (150 nM) was added to half the vials at *t*=0 hr. For these conditions complete removal of N_2_O was expected in 5 min when N_2_O Reductase was present, see Supporting Information and Figure S6 for further details.PLEASE REPLACE THE EXISTING TOC IMAGE with the one below.

Gas chromatography was used to assess photocatalytic removal of N_2_O by our liposome microreactors (Figure 5A). Headspace N_2_O concentration was sampled over 8 hr, with white‐light irradiation in the first 4 hr, for suspensions of proteoliposomes containing MV, N_2_O, graphitic N‐doped carbon dots and 25 mM EDTA. The headspace N_2_O concentration dropped significantly only for those proteoliposomes that included MtrCAB (Figure 5A, Figure S6A). This behavior continued in the dark because, even after irradiation, N_2_O continued to transfer from the head space gas to the reaction liquid due to a slow gas exchange between these phases as described below (Figure 5B, Figure S6B).

The integrity of the liposome bilayers during light driven N_2_O removal was evident from control experiments which revealed that the activity of N_2_O Reductase, when free in solution, was lowered 15× by incubation with 25 mM EDTA for 10 min, e.g., Figure S3B. We expected further loss of function over time and attribute this to chelation of the copper‐cofactors essential for enzyme function or the calcium ions that stabilize the dimer interface.^[22]^ Thus, a key role of the liposomal membrane during light driven N_2_O reduction is the separation of the aqueous redox compartments and mimicry of the design principles of natural photosynthesis such that the sacrificial electron source EDTA does not contact internal N_2_O Reductase.

Gas chromatography provided direct evidence for N_2_O reduction by internalized enzyme and supports the primary role of MtrCAB in trans‐membrane electron transfer. The slower rate of N_2_O removal by irradiated carbon dots than dithionite can be attributed to two factors. Firstly, slow partitioning of N_2_O between liquid and gas phases (Figure 5B, Figure S6B). Secondly, a steady, slower supply of photoexcited electrons than the essentially immediate electron release possible with dithionite.

Regarding the rate limiting events associated with intrinsic microreactor components, the dithionite driven steady state catalytic rate in the MtrCAB containing liposomes is comparable to that of lysed proteoliposomes, approximately 1 nmol N_2_O reduced min^−1^ (μL liposome stock)^−1^. This observation is consistent with N_2_O reduction providing the limiting step in microreactor performance. Indeed, without a microreactor the maximum turnover frequency (*k*_cat_) for the N_2_O Reductase used in these experiments was determined to be approximately 20 s^−1^ (see Experimental Section, Supporting Information) and several orders of magnitude lower than the trans‐membrane electron flux^[7a]^ that can be supported by MtrCAB. From the rate of dithionite oxidation reported for the liposomes in this study, together with the spectroscopically defined MtrCAB concentration in the sample (see above), an electron flux of 28 s^−1^ MtrCAB^−1^ is estimated which is again consistent with a rate limiting step associated with catalysis by N_2_O Reductase. During photocatalytic N_2_O removal, similar electron transfer processes and rate‐limiting steps are anticipated since irradiated graphitic N‐doped carbon dots display facile reduction of MV^2+[20]^ and of MtrCAB in lipid bilayers.[^15^, ^21^]

## Conclusion

Respiration and photosynthesis use lipid bilayers to arrange and spatially separate redox proteins of different functionality so that trans‐membrane electron transfer can harness energy for ATP synthesis and the reduction of NAD(P)^+^. We have mimicked that physical separation in this work to drive electrons across an insulating lipid bilayer membrane through MtrCAB electron conduits to encapsulated N_2_O Reductase that converts the potent greenhouse and ozone‐reactive gas N_2_O to the climate‐neutral product N_2_. The liposomal microreactors perform photocatalytic N_2_O removal under ambient conditions using only earth abundant elements. In addition, we note that MV is well‐known as a general electron donor, capable of driving reductive catalysis by numerous redox enzymes and synthetic catalysts. Thus, the microreactor design, with redox cycling of encapsulated MV to transfer electrons from MtrCAB to internalized catalysts, opens the door to creating bespoke systems tuned to perform different reaction cascades through the activities of multiple enzymes and/or synthetic catalysts harnessing energy from external light‐harvesting particles.

## Conflict of interest

The authors declare no conflict of interest.

1

## Supporting information

As a service to our authors and readers, this journal provides supporting information supplied by the authors. Such materials are peer reviewed and may be re‐organized for online delivery, but are not copy‐edited or typeset. Technical support issues arising from supporting information (other than missing files) should be addressed to the authors.

Supporting Information

## Data Availability

The data that support the findings of this study are openly available in figshare at https://doi.org/10.6084/m9.figshare.20337783, reference number 20337783.
